# Are We Predicting the Actual or Apparent Distribution of Temperate Marine Fishes?

**DOI:** 10.1371/journal.pone.0034558

**Published:** 2012-04-19

**Authors:** Jacquomo Monk, Daniel Ierodiaconou, Euan Harvey, Alex Rattray, Vincent L. Versace

**Affiliations:** 1 School of Life and Environmental Sciences, Faculty of Science and Technology, Deakin University, Warrnambool, Victoria, Australia; 2 School of Plant Biology (Oceans Institute M470), The University of Western Australia, Crawley, Western Australia, Australia; 3 School of Information Systems, Faculty of Business and Law, Deakin University, Warrnambool, Victoria, Australia; Victoria University Wellington, New Zealand

## Abstract

Planning for resilience is the focus of many marine conservation programs and initiatives. These efforts aim to inform conservation strategies for marine regions to ensure they have inbuilt capacity to retain biological diversity and ecological function in the face of global environmental change – particularly changes in climate and resource exploitation. In the absence of direct biological and ecological information for many marine species, scientists are increasingly using spatially-explicit, predictive-modeling approaches. Through the improved access to multibeam sonar and underwater video technology these models provide spatial predictions of the most suitable regions for an organism at resolutions previously not possible. However, sensible-looking, well-performing models can provide very different predictions of distribution depending on which occurrence dataset is used. To examine this, we construct species distribution models for nine temperate marine sedentary fishes for a 25.7 km^2^ study region off the coast of southeastern Australia. We use generalized linear model (GLM), generalized additive model (GAM) and maximum entropy (MAXENT) to build models based on co-located occurrence datasets derived from two underwater video methods (i.e. baited and towed video) and fine-scale multibeam sonar based seafloor habitat variables. Overall, this study found that the choice of modeling approach did not considerably influence the prediction of distributions based on the same occurrence dataset. However, greater dissimilarity between model predictions was observed across the nine fish taxa when the two occurrence datasets were compared (relative to models based on the same dataset). Based on these results it is difficult to draw any general trends in regards to which video method provides more reliable occurrence datasets. Nonetheless, we suggest predictions reflecting the species apparent distribution (i.e. a combination of species distribution and the probability of detecting it). Consequently, we also encourage researchers and marine managers to carefully interpret model predictions.

## Introduction

Worldwide human activity is having adverse impacts on the structure and function of marine ecosystems [1]. In response, many initiatives are underway to identify, prioritize and ultimately preserve areas of importance [2], [3], [4], [5], [6]. An initial step in this process often involves delineating the distribution of species, assemblages or habitats [7]. This allows areas that support high diversity to be given the highest priority, which is particularly important when the maintenance and enhancement of biodiversity is the central goal of a management initiative [8]. To support such strategies, management agencies are increasingly seeking the provision of accurate, quantitative and spatially-explicit information on patterns of species distributions at scales relevant to the assessment and management process [3], [9].

In this context, predictive modeling of species’ distribution has become a fundamental tool [7]. These models have provided a popular analytical framework for relating geo-located observations of occurrence to environmental variables that contribute to a species distribution [7]. This relationship is based on statistically or theoretically derived response functions that characterize the environmental conditions associated with the ecological niche of a given organism [10]. Presence/absence models are frequently used to predict species distributions, but there is a common problem related to uncertainty in determining absences [11]; especially where the species is difficult to survey and does not appear to occupy all available suitable habitats [12]. In such cases, researchers have two options; (1) model presence/pseudo-absence (or background) data (e.g. [13]), or (2) model presence-only data (e.g. [14]). However, the use of a random sample from the background population to supply pseudo-absences may have unexpected consequences on results when true absences are expected [15], [16]. For example, Wisz and Guisan [15] suggested that models built using random pseudo-absence datasets are expected to have lower predictive performance than models built with actual absences. In fact, it may be argued that, on a theoretical basis at least, a presence-only approach may be preferable because there is no requirement for truly exhaustive and exclusive absences; a requirement that is not met by most biodiversity data.

Often in the marine environment species distribution models (SDMs) are based on occurrence data collected by the researcher. This often results in predictions that are reasonable depictions of the focal species distribution. While there are many different methods available to provide occurrence datasets for demersal fishes in the marine environment, baited and towed systems are being used as they overcome many issues associated with traditional survey methods (for a review of these issues see [17]). Moore et al. [18], for example, modeled temperate marine fish distributions based on baited-video-derived occurrence and fine-scale multibeam sonar datasets using classification trees and generalized additive models (GAMs). Similarly, Stoner et al. [19] used towed-video observations to model the relationships between *Lepidopsetta polyxystra* (northern rock sole) abundance and environmental variables (e.g. depth, sediment qualities, macroalgae) in five near-shore nursery grounds. The value of these data is not questioned; however the representativeness of these occurrence localities is dependent on which survey technique is used. For example, a comparison between baited and unbaited video, found that a greater number of individuals and species were recorded by the baited system; especially carnivorous fishes [20]. While research suggests that deploying a combination of survey methods used concurrently provides a better assessment of fish assemblages (e.g. [21]), the logistical or financial constraints of most studies limit fish biodiversity assessments to only one method. Consequently, understanding the influence of a survey method used to collect occurrence datasets for modeling of fine-scale habitat suitability is especially crucial. If an observation of zero individuals has arisen because it was present but not detected then any statistical inference based on such data are likely to be incomplete or wrong [22]. Consider a fish species that was observed in 10% of a study region. This fish may actually occur throughout the entire area but was only detected 10% of the time. Alternatively, it may also be found in only 10% of the area and has been detected perfectly.

Consequently, the aim of this paper is to highlight the potential differences in model predictions caused by the choice of survey method used to collect fish occurrence datasets. In this paper towed and baited underwater video methods are used to provide occurrence datasets for nine temperate marine sedentary fishes. Further, given the potential for presence/absence and presence-only models to produce considerably different predictions of habitat suitability, three commonly applied SDMs (i.e. generalized linear models; GLMs, GAMs and maximum entropy; MAXENT) are applied to the baited and towed video occurrences datasets for the nine fish taxa. This will provide a comparison of survey method as well as determine how these datasets potentially influence both types of SDMs (i.e. presence/absence and presence-only). With increasing application of SDMs in the marine environment, this paper will bring into focus the need for careful interpretation of predictions.

## Results

### Model Evaluation

The Area Under the receiver-operating characteristic Curves (AUC) was used to evaluate the models of habitat suitability for the nine demersal fish taxa. All of the 54 models of habitat suitability returned AUC values >0.5 (Table 1). Of these, MAXENT provided the top 12 highest performing models as measured by AUC. On 15 occasions GLMs and GAMs produced the same performing models as each other (as measured by AUC); with four of these being the equal highest performing model. In isolation, GLMs and GAMs only provided one model each that performed highest.

**Table 1:** Summary of model performances as measured by AUC for the baited and towed video datasets.

Taxon	Observation technique	GAM AUC	GLM AUC	MAXENT AUC
*Caesioperca* spp.	Baited video	0.73	0.80	0.63
	Towed video	0.57	0.57	0.61
*Cheilodactylus nigripes*	Baited video	0.66	0.66	0.65
	Towed video	0.77	0.77	0.84
*Meuschenia scaber*	Baited video	0.96	0.96	0.92
	Towed video	0.69	0.59	0.68
*Notolabrus tetricus*	Baited video	0.89	0.89	0.90
	Towed video	0.75	0.75	0.84
*Odax cyanomelas*	Baited video	0.87	0.87	0.92
	Towed video	0.81	0.75	0.90
*Parequula melbournensis*	Baited video	0.70	0.70	0.82
	Towed video	0.67	0.67	0.59
*Pempheris multiradiata*	Baited video	0.82	0.82	0.89
	Towed video	0.70	0.70	0.73
*Pseudolabrus psittaculus*	Baited video	0.90	0.90	0.87
	Towed video	0.60	0.60	0.68
*Upeneichthys vlamingii*	Baited video	0.72	0.72	0.74
	Towed video	0.54	0.54	0.63

### Similarity in Predictions of Habitat Suitability

The similarity between predictions of habitat suitability for the nine demersal fish taxa from the two observation techniques was assessed using the *I* statistic [23]. These *I* values were grouped by thresholds adapted from Roubicek et al. [24], which indicated *I* values <0.7 as low, 0.7–0.8 moderate, and >0.8 as highly degree of similarity between predictions of habitat suitability.

#### Similarity in habitat suitability models derived from baited-video

**Figure 1:**
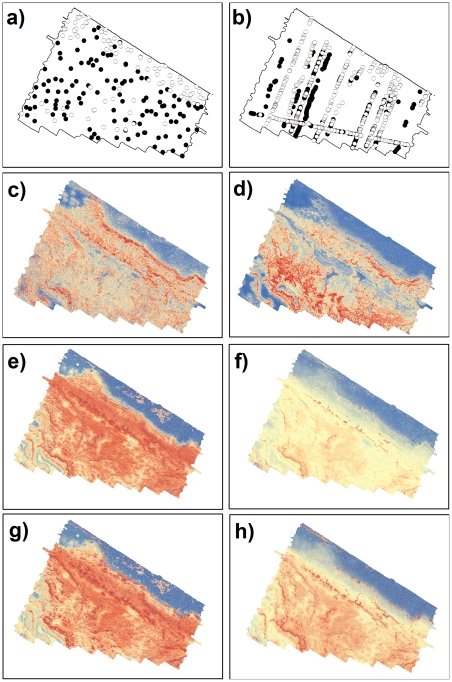
Example of similar habitat suitability predictions. Example of predicted habitat suitability for *Caesioperca* spp. showing very similar predictions based on the baited and towed video datasets. Left column: baited video. Right column: towed video. (a–b) presence/pseudo-absence localities (presence: black; pseudo-absence: white). (c–d) MAXENT predictions. (e–f) GLM predictions (g–h) GAM predictions. Red shading indicates high suitability, while blue highlights low suitability.

**Figure 2:**
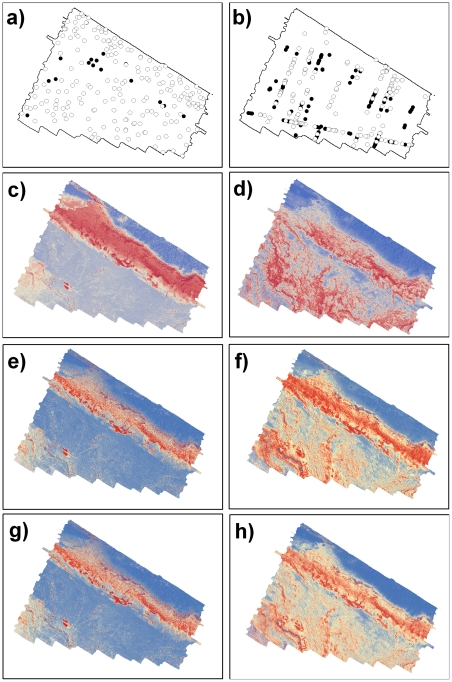
Example of dissimilar habitat suitability predictions. Example of predicted habitat suitability for *Pempheris multiradiata* showing dissimilar predictions based on the baited and towed video datasets. Left column: baited video. Right column: towed video. (a–b) presence/pseudo-absence localities (presence: black; pseudo-absence: white). (c–d) MAXENT predictions. (e–f) GLM predictions (g–h) GAM predictions. Red shading indicates high suitability, while blue highlights low suitability.

Generally, all three modeling approaches provided predictions with a high degree of similarity for the nine focal fish taxa based on the baited-video dataset (i.e. I > 0.80; Table 2; Figure 1; Figure 2). The GAMs and GLMs provided very similar model predictions (i.e. I ∼ 1) for all nine fish taxa. Only slight differences were observed between MAXENT and the other two modeling approaches; with the greatest difference being observed for predictions of habitat suitability for Pempheris multiradiata (common bullseye; Table 2; Figure 2).

**Table 2:** Summaries of the similarity between habitat suitability predictions using the *I-*statistic (*I* ≈ 1: identical, *I* ≈ 0: completely different).

			Baited			Towed	
			GAM	GLM	MAXENT	GAM	GLM
*Caesioperca* spp.	Baited	GLM	0.96				
		MAXENT	0.87	0.88			
	Towed	GAM	0.92	0.93	0.88		
		GLM	0.92	0.93	0.88	1.00	
		MAXENT	0.87	0.87	0.83	0.87	0.87
*Cheilodactylus nigripes*	Baited	GLM	0.99				
		MAXENT	0.82	0.82			
	Towed	GAM	0.76	0.76	0.83		
		GLM	0.76	0.76	0.83	1.00	
		MAXENT	0.76	0.76	0.82	0.90	0.90
*Meuschenia scaber*	Baited	GLM	0.98				
		MAXENT	0.90	0.90			
	Towed	GAM	1.00	1.00	0.75		
		GLM	0.75	0.75	0.82	0.80	
		MAXENT	0.82	0.82	0.64	0.66	0.60
*Notolabrus tetricus*	Baited	GLM	0.95				
		MAXENT	0.90	0.90			
	Towed	GAM	0.87	0.87	0.84		
		GLM	0.87	0.87	0.84	1.00	
		MAXENT	0.79	0.79	0.77	0.86	0.86
*Odax* *cyanomelas*	Baited	GLM	0.99				
		MAXENT	0.89	0.89			
	Towed	GAM	0.81	0.81	0.80		
		GLM	0.76	0.76	0.75	0.81	
		MAXENT	0.82	0.82	0.81	0.83	0.76
*Parequula melbournensis*	Baited	GLM	0.98				
		MAXENT	0.86	0.86			
	Towed	GAM	0.68	0.68	0.70		
		GLM	0.68	0.68	0.70	1.00	
		MAXENT	0.82	0.82	0.87	0.72	0.72
*Pempheris multiradiata*	Baited	GLM	0.95				
		MAXENT	0.77	0.77			
	Towed	GAM	0.63	0.63	0.67		
		GLM	0.63	0.63	0.67	0.97	
		MAXENT	0.60	0.60	0.70	0.83	0.83
*Pseudolabrus psittaculus*	Baited	GLM	0.97				
		MAXENT	0.87	0.88			
	Towed	GAM	0.75	0.75	0.75		
		GLM	0.75	0.75	0.75	0.89	
		MAXENT	0.71	0.71	0.70	0.89	0.89
*Upeneichthys vlamingii*	Baited	GLM	0.99				
		MAXENT	0.87	0.87			
	Towed	GAM	0.78	0.78	0.79		
		GLM	0.78	0.78	0.79	0.78	
		MAXENT	0.80	0.80	0.82	0.78	1.00

#### Similarity in habitat suitability models derived from towed-video

When compared to the baited-video datasets, more variation in similarity between the three modeling approaches was observed across the nine fish taxa based on the towed-video datasets (Table 2; Figure 1; Figure 2). Similar to the baited-video-derived models, GAM and GLM provided identical or very similar predictions for eight of the nine taxa; with only moderate differences being observed for *Upeneichthys vlamingii* (southern goatfish; *I*  =  0.78). Similarly, comparison between MAXENT and the other two modeling approaches showed a high degree of similarity for five of the nine fish taxa. For *U. vlamingii* GAM was moderately different to both GLM and MAXENT, but no difference was observed between GLM and MAXENT (*I*  =  1). Similarly, both GAM and GLM were moderately different to MAXENT for *Parequula melbournensis* (silverbelly), but no difference was observed between GAM and GLM (Table 2). *Meuschenia scaber* (cosmopolitan leatherjacket) showed the same trend as *P. melbournensis*, albeit to a greater degree (i.e. low similarity; Table 2). The GLM and MAXENT predictions showed only moderate similarity for *Odax cyanomelas* (herring cale).

#### Similarity in habitat suitability models between observation techniques

In contrast to models derived from the same dataset (i.e. baited or towed video), greater dissimilarity were observed when the predictions of habitat suitability based on the two video observation techniques were compared (Table 2). *Caesioperca* spp. (perch) was the only taxa to exhibit a high degree similarity for all modeling approaches between the two observation datasets (Table 2; Figure 1). With exception of the baited-video-derived MAXENT model for *Cheilodactylus nigripes* (magpie morwong) that showed a high degree of similarity with towed-video-derived MAXENT, models for this species had a moderate degree of similarity between observation datasets irrespective of modeling approach (Table 2). Identical predictions of habitat suitability were observed for GAM and GLM between observation datasets for *M. scaber* (Table 2). A high degree of similarity was observed for towed-video-derived MAXENT and baited-video-derived GAM and GLM (Table 2). Towed-video-derived GLM also showed a high degree of similarity with baited-video-derived MAXENT. A moderate degree of similarity was also observed between the towed-video-derived GLM and the baited-video-derived MAXENT. However, the towed-video-derived MAXENT showed a low degree of similarity with baited-video-derived MAXENT. For *Notolabrus tetricus* (blue-throated wrasse) a high degree of similarity was observed between the two observation datasets for GAM, GLM and MAXENT. Towed-video-derived MAXENT, however, showed a moderate degree of similarity with baited-video-derived GAM, GLM and MAXENT (Table 2). Predictions of habitat suitability using GAM, GLM and MAXENT based on either observation technique showed a high degree of similarity for *O. cyanomelas*. Towed-video-derived GLM, however, showed a moderate degree of similarity with all three models based on baited-video data. Towed-video-derived MAXENT for *P. melbournensis* showed a high degree of similarity with all three baited-video-derived models. Both towed-video-derived GAM and GLM showed the same trend; with a low degree of similarity between GAM and GLM between observation datasets (Table 2). Baited-video-derived MAXENT showed a moderate degree of similarity (i.e. *I*  =  0.70). A moderate degree of similarity between the two observation datasets was observed for all models for *Pseudolabrus psittaculus* (rosy wrasse; Table 2). Towed-video-derived MAXENT for *U. vlamingii* showed a high degree of similarity with all three baited-video-derived models. In contrast, GAM and GLM showed a moderate degree of similarity with all three baited-video-derived models (Table 2). A low degree of similarity between the two observation datasets was observed for all models for *P. multiradiata* (Table 2; Figure 2).

## Discussion

This study explored two commonly used underwater video techniques to provide occurrence data to develop and compare presence/pseudo-absence and presence-only fine-scale, habitat suitability models for nine species of temperate marine sedentary fishes. The habitat suitability models built in this study performed considerably better than random when assessed by AUC. The AUC values recorded in this study are similar to those observed in previous marine and terrestrial habitat suitability modeling studies (e.g. [14], [25], [26], [27]). Despite the fact that AUC has recently been criticized (see [28], [29]), it does provide a preliminary indication of the usefulness of a model for the identification of suitable habitat for a particular species [14]. This study has also demonstrated this to be the case, and further, that baited and towed video survey techniques are capable of providing models of similar quality (AUC); a conclusion supported by other recent studies [18], [25], [30], [31], [32], [33]. For example, Moore et al. [18] compared the ability of presence/absence methods (GAM and classification and regression trees) to predict fine-scale habitat suitability for demersal fishes based on baited-video and multibeam sonar datasets. They found that baited-video and multibeam sonar datasets were useful in providing a detailed understanding of demersal fish-habitat associations, as well as accurately predicting species distributions across unsurveyed locations where continuous spatial seafloor data were available [18]. Similarly, Monk et al. [25] used towed-video and multibeam sonar derived datasets to compare commonly used presence-only methods (i.e. BIOCLIM, DOMAIN, Ecological-Niche Factor Analysis, MAXENT). They concluded that towed-video-based occurrence data provided well-performing, fine-scale models and encouraged the ongoing use of presence-only approaches, particularly MAXENT, in modeling suitable habitat for demersal marine fishes [25]. Despite these studies supporting the idea that underwater-video-based occurrence and multibeam sonar-derived datasets are capable of providing well-performing models, this study is one of the first to contrast these two video observation techniques for generating occurrence datasets for predictions of fine-scale habitat suitability for temperate marine fishes (using both presence/pseudo-absence and presence-only modeling techniques).

While numerous studies have compared modeling approaches in terms of model performance (i.e. via AUC or kappa; [14], [25], [26], [27], [34], [35]), the main purpose of this study was to highlight how sensible-looking, well-performing (based on AUC) models can provide very different predictions of habitat suitability depending on which video observation dataset was used. Overall, greater dissimilarity between the three modeling approaches was observed across the nine fish taxa when models based on the two occurrence datasets were compared (relative to models based on the same survey method). This finding suggests that the characteristics of the occurrence data are important. This concept is supported by Kadmon et al. [36] who suggested that models are influenced by the reliability of occurrence data and distribution characteristics of the modeled species. The latter has been thoroughly discussed in previous marine and terrestrial studies (e.g. [25], [26], [36], [37]), and suggest that narrowly distributed species that exhibit minimal niche variation provide more reliable models.

There are many factors that potentially influence the reliability of occurrence data of fishes, including traits such as; body size, crypticity, schooling behavior, habitat, camera avoidance and observer biases [38], [39], [40]. These issues are all inherently linked to the choice of survey method. For example, on numerous occasions, males of *N. tetricus* were observed aggressively guarding the bait against conspecific males and, in some cases, other species. Guarding behavior from territorial species could potentially lead to underestimates of species and densities on deployments where it is occurring [41]. Similarly, after reviewing some of the baited-video footage where the deployment vessel is heard approaching (to retrieve the unit after the 60-min deployment), it was noticed that on several occasions the fishes in the field of view rapidly departed. Avoidance behavior towards boat noise could potentially bias the towed-video dataset and has been reported by other researchers (e.g. [42], [43]). Sarà et al. [42] found that agonistic behavior of *Thunnus thynnus* (northern bluefin tuna) was more evident when exposed to sounds from outboard motors. Further, Stoner et al. [44] found that the presence of underwater camera systems (e.g. towed-video system or remotely operated vehicles) could potentially bias the fish species observed; albeit difficult to quantify as most avoidance (or attraction) occurs outside the field of view.

Another possible explanation for the disparity between predictions of habitat suitability may be attributed to the deployment differences between the surveying methods (i.e. stationary *v.* moving). Consider habitat patches with comparable fish population density but varying in natural shelters such as crevices or macroalgae. Sampling that relies on the use of a moving platform (e.g. an obliquely angled towed-video camera that is flown 2 m above the seafloor) to provide visual observations could result in incomplete detection in habitat patches with more natural cavities or canopy forming macroalgae (e.g. kelp). By contrast, sampling that relies on a stationary platform (e.g. baited-video) to provide a visual observation dataset may allow time for species that are hiding amongst the crevices or canopy forming macroalgae to be observed. However, the use of bait in these systems is well documented to increase the number of pelagic or epibenthic carnivores in the vicinity of the camera deployment [45], [46], [47], which may result in reduced observations of cryptic or prey species recorded by these systems. The deployment of unbaited stationary cameras may decouple the effects of bait and seafloor structure, and warrants further investigation.

While the use of presence-only methods (such as MAXENT in this study) potentially reduces the issue of non-detections, the fact still remains that if a species is less detectable in a subset of its niche by a particular survey method, then this may influence model predictions. As highlighted by the differences between MAXENT model predictions observed in this study, the two video survey methods detected presences in slightly different environmental niches. For example, the fish taxon that provided predictions that were most similar was *Caesioperca* spp. (Figure 1). These are conspicuous aggregating fish species that are commonly observed in cloud-like schools feeding above reef crests [48]. Consequently, both video methods detected this conspicuous species in the same ecological niche, and are thus reflected in the similar predictions of suitable habitat. Conversely, *P. multiradiata* showed the lowest similarity in model predictions between survey methods (Figure 2). This timid species inhabits caves ranging from shallow (∼10 m) macroalgal-dominated reefs to deeper (∼50 m) invertebrate colonized systems [49]. For this species, the two video methods detected individuals in different environmental niches. For example, the stationary characteristic of the baited-video method enabled individuals hiding among the shallow, complex reef systems to be recorded. However, the baited system recorded fewer occurrences in the deeper regions, which can possibly be attributed to the higher number of pelagic and epibenthic carnivores that were attracted to (and recorded by) the baited-video throughout these areas. This may result in avoidance by *P. multiradiata* from the baited-video on deployments were these predatory fishes were present in high numbers. By contrast, the towed video did not attract these predatory individuals, and recorded more *P. multiradiata* in the deeper invertebrate dominated regions of the study site.

Whilst all model predictions for the nine fish taxa reflect aspects of their known ecology, the results from this study suggest that differences between model predictions are actually reflecting the apparent species distribution (i.e. a combination of the habitat suitability of a fish species and the probability of detecting it; [50]). This bias cannot be resolved without consideration of variations in detectability that may arise from differences between survey methods, habitats and species [51]. Additionally, the strength of a habitat variable relationship in a SDM may be underestimated whenever imperfect detection is not accounted for, even with constant detectability [52]. Although some conventional SDMs allow for the problem of missing non-detection data to be partially addressed (i.e. missing zeros; [53]) or permit very general functional forms of covariates to be fitted, such as regression trees [54] and boosted regression trees [55], site-occupancy models may provide a useful alternative [50]. Site-occupancy models use the presence/absence (or more correctly termed detection/non-detection) patterns at sites surveyed multiple times (i.e. at least twice) to separate the sampling method from the ecological process and thus obtain estimates of the true species distribution along with unbiased estimates of variable importance [50], [52], [56]. However, temperate marine fish studies have rarely addressed the issue of detectability in video-derived occurrence datasets as surveying a site (especially for the purpose of building localized, fine-scale SDMs) more than once is often impractical due to limited weather windows (for more multiple surveys in a single field season) and deployment costs (both in a single season and between seasons). While underwater visual census methods have developed distance sampling techniques (e.g. [57]) that enable detectability to be accounted for, further research is needed to determine the relative detectability of fishes using towed or baited video systems.

### Conclusions

This study has demonstrated that the characteristics of the video-derived occurrence data are potentially more important than the chosen modeling technique in developing fine-scale models of habitat suitability for temperate marine fishes. However, based on the results in the present study it is difficult to draw any general trends in regards to which circumstances what survey method provides more reliable occurrence datasets. Nonetheless, the main objective here was not to directly compare model performance, or even emphasize which of the two video methods compared is better for building models of habitat suitability for marine fishes. Instead, the purpose of this paper was to raise awareness that interpretation of habitat suitability models needs to account for the potential influence that the choice of survey method used to provide occurrence datasets may have. Whilst limitations within the datasets used in the present study precluded the use of site-occupancy models, which incorporate measures of detectability, it is suggested that these models may provide a practical alternative to conventional SDMs to predict the distribution of suitable habitat for demersal fishes. However, in the absence of repeat surveys (which would enable the use of enable site-occupancy models), researchers should select the method that is most likely to best detect their focal species; given the known behavior and ecology of the species (e.g. will the species react to bait? Or will the species respond to a moving camera?). Alternatively, conventional SDMs could be built utilizing occurrence datasets derived from different survey methods deployed at the same study site in a single field season. The similarity between these predictions can then be assessed (e.g. using *I-*statistic) to ensure that model outputs reflect as close to the actual distribution of suitable habitat for marine fishes as possible.

## Materials and Methods

### Study Site

**Figure 3:**
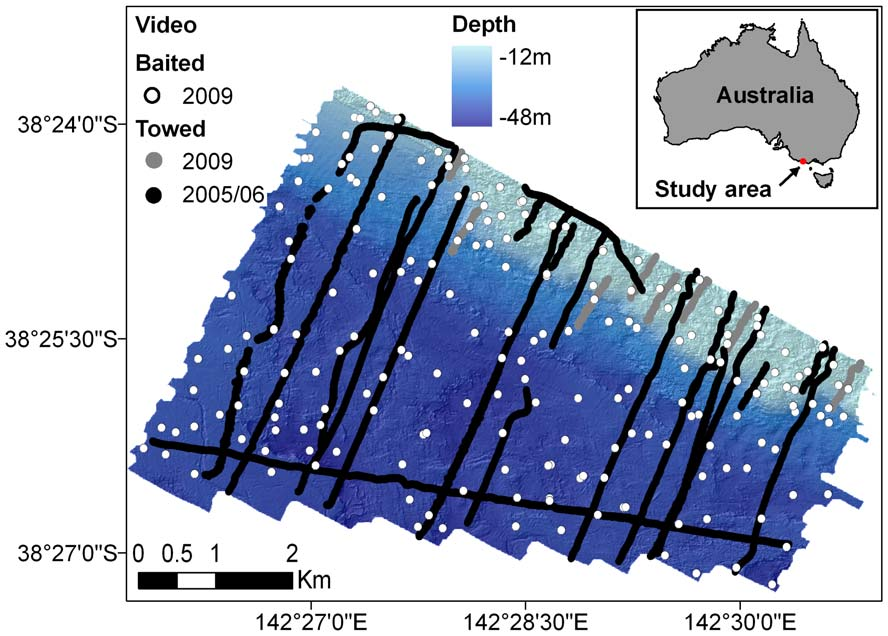
Study area. The location of the Warrnambool study area off the south-eastern coast of Australia. Shading indicates water depth. Black lines indicate towed video transects. White dots indicate baited video deployments. Red line delineates the southern extent of the Hopkins Bank.

The study site encompassed an area 25.7 km^2^ that was situated offshore from the city of Warrnambool (38° 43′ S, 142° 43′ E), south-eastern Australia (Figure 3). The site ranged in depth from 12 to 50 m (calculated from multibeam sonar coverage for the study area which is based on lowest astronomical tide datum). The deeper regions consisted of a mixture of low (<1 m) profile reef and sandy sediments dominated by mixed red algae, sponges, ascidians, bryozoans and gorgonian corals [58]. The shallow reefs along the northern section (Hopkins Bank) of the study site were dominated by canopy-forming kelp (*Ecklonia radiata* and *Phyllospora comosa*). A large sandy area in the north-western region of the study site also supported a sparse cover of seagrass (Zosteraceae).

### Fish Surveying Techniques

#### Ethics statement

This study was undertaken in strict accordance with the recommendations and procedures determined by the Prevention of Cruelty to Animals Act 1986 and its Regulations, and the Australian Code of Practice for the Care and Use of Animals for Scientific Purposes. This protocol was approved by the Deakin University Animal Welfare Committee (permit number A9-2009).

#### Video deployments

Two video methods were deployed; (1) baited and (2) towed. The sampling strategy for each method was designed to provide adequate spatial coverage of the study site and to be representative of the dominant substrata types and benthic biological habitats, whilst also being appropriate for SDM analysis.

#### Baited-video deployments

The sampling strategy for the baited-video drops was a stratified random design. Stratified deployments were allocated utilizing the multibeam sonar bathymetry and accurately predicted biotic habitat map available for the study area. The validation test for this biota map returned overall accuracy of 83%. Detailed descriptions of the method used to validate the biota map are available in Ierodiaconou et al. [58]. Ten replicate drops were preformed across three multibeam sonar derived variables; (1) bathymetry was grouped into 10 m depth strata (i.e. 10–19, 20–29, 30–39 and 40–49 m), (2) rugosity was reclassified into high, medium and low strata and (3) Benthic Position Index (BPI) was classed into trough, flat and peak. For predicted biotic habitat, 10 replicate drops were performed in each of the six predicted habitats (e.g. mixed brown algae, for details see; [58]). To ensure adequate spatial coverage 59 additional drops were randomly allocated throughout the deeper (i.e. >20 m) regions of the study site.

The baited-video systems used comprised two Sony HC 15E video cameras mounted 0.7 m apart on a base bar inwardly converged at 8° to gain an optimized field of view with visibility of ∼7 m distance (water clarity dependent; [59]). Detailed information on the design and photogrammetric specifics can be viewed in Harvey and Shortis [60]. Up to five baited-video systems were deployed at any one time to increase sampling efficiency. Each baited-video system was deployed by boat and left to film on the seafloor for a period of 1 h. At least 36 min of filming time is recommended to obtain measures for the majority of fish species, though 60 min is advisable to obtain measures of numerous targeted fish species [61]. Each camera system was equipped with a synchronizing Light Emitting Diode (LED) that was visible in the fields of view of both video cameras. The LED was used to check synchronization of the video footage, thereby eliminating systematic error of motion parallax [59]. The LED emitted minimal light and was standard across all drops. Each system was also baited with ∼800 grams of crushed *Sardinops sagax* (pilchard) in a closed plastic-coated wire mesh basket, suspended 1.2 m in front of the two cameras. Adjacent concurrent drops were separated by at least 250 m to avoid overlap of bait plumes and reduce the likelihood of fish moving between sites within the 1 h sampling period [62]. This distance is well accepted by baited camera operators to reduce the effects of bait plume between concurrent deployments [20], [62], [63], [64]. All drops were deployed between 08∶00 and 18∶00 to minimize the effects of diurnal changes in fish behavior [65].

Fish were sampled from February to March 2009, with a total of 219 60-min baited-video deployments precisely positioned with a differential GPS to ensure accurate spatial location (Figure 3). However, 15 deployments were excluded from the analysis due poor visibility or being smothered in kelp. Footage from each baited-video deployment was interrogated to obtain the maximum number of fish belonging to each species present in the field of view of the cameras at one time (MaxN; [66]). These observations were obtained using the program EventMeasure (SeaGIS Pty Ltd; www.seagis.com.au) and converted to presence/pseudo-absence and presence-only datasets (see ‘*Data Treatment*’).

#### Towed-video transects

Twenty nine towed-video transects were used to provide fish occurrence data. The 29 transects were selected to encompass the main physical gradients of the study site (e.g. depth, topographic variation, exposure). Additional transects were undertaken throughout the shallow heterogeneous regions of the site to ensure adequate representation of habitats throughout the study site. The 29 transects covered 68 linear km of the study area (Figure 3). Over eight days (December-March 2005/06 and February-March 2009) a micro remotely-operated vehicle (VideoRay Pro 3) was towed along the 29 transects at 0.5−1 ms^−1^ (1–2 knots). The oblique angled camera was maintained approximately 2 m from the bottom with a field of view of ∼7 m distance (water clarity dependent). The distance from the seafloor was monitored and maintained using live-feed video and a vessel-mounted winch system. A text overlay containing a time stamp and transect ID were recorded with the video using a Sony MiniDV recorder. The video footage was interrogated to identify fish species and the spatial position of each occurrence locality was then determined by matching the time stamp of the video with the corresponding survey positional data. The towed-video system was geo-located by integrating vessel location (Omnistar satellite dGPS), motion sensor (KVH) and acoustic camera positioning (Tracklink Ultra Short Baseline). The total propagated error at the maximum depth of the study site was ±5 m accuracy.

### Species Distribution Models

The SDMs were built for the nine most common fish taxa (Table 3) across the two video survey techniques. For each of the fish taxon, GLM, GAM and MAXENT models were built using the same training and evaluation data derived from either the baited and towed video datasets. By using the same training and evaluation dataset derived from baited and towed video model performance can be directly compared.

**Table 3:** Summary of the number of occurrences used in model building for each taxon based on the two video methods.

Taxon	Video method	Presence	Pseudo-absence
*Caesioperca* spp.	Baited	115	87
	Towed	431	431
*Cheilodactylus nigripes*	Baited	38	164
	Towed	32	32
*Meuschenia scaber*	Baited	106	96
	Towed	37	37
*Notolabrus tetricus*	Baited	114	88
	Towed	50	50
*Odax cyanomelas*	Baited	39	163
	Towed	56	56
*Parequula melbournensis*	Baited	29	173
	Towed	15	15
*Pempheris multiradiata*	Baited	15	187
	Towed	154	154
*Pseudolabrus psittaculus*	Baited	61	141
	Towed	90	90
*Upeneichthys vlamingii*	Baited	37	165
	Towed	31	31

#### Generalized linear models

The GLM is often used in ecological studies, and therefore serves as a benchmark for the other model types [67]. The GLMs were built in the Marine Geospatial Ecology Tool kit (MGET; Duke University), which interfaces between statistical software ‘*R*’ (and its contributing packages; [68]) and ArcGIS 9.3. Each fish taxon was individually modeled using a logit link and a binomial error term. All models were fitted with the predictor variables listed in Table 4 using a backward stepwise procedure. The Akaike Information Criterion (AIC) was used to determine variable contribution as predictor variables were sequentially added and then dropped from the model.

**Table 4:** Description of the nine seafloor variables retained to model building.

Variables	Variable description	Software
*Aspect*-Eastness Northness	Aspect (azimuthal bearing of steepest slope) has a inherent circularity built in, to overcomethis, two trigonometric transformations [60] were applied; northness (sin(aspect)) and eastness(cos(aspect)). These two variables represent proxies for exposure.	Spatial Analyst- ArcGIS 9.3
Bathymetry	Bathymetry provides a measure of water depth based on lowest astronomical tide datum.	Fugro Starfix suite 9.1
Benthic position index	Measure of a location relative to the overall landscape. Calculated by comparing the elevationof a cell with the mean elevation of surrounding cells by the three analysis extents. Regions with positive values are higher than their surroundings, whereas areas negative values are lower. Flatareas have values closer to zero [61]	Benthic Terrain Modeler Tool for ArcGIS
Euclidean distance to bank	Hopkins bank is a major reef feature along the north section of the study region. This bankfeature was extracted from a predicted reef class from a substratum map that was generated using a decision tree classifier [45]. The Euclidean distance to this feature was calculated in meters.	Spatial Analyst- ArcGIS 9.3
Euclidean distance to nearest reef	A predicted reef class from a substratum map, generated using a decision tree classifier [45],was used to calculate Euclidean distance (in meters) to nearest reef.	Spatial Analyst- ArcGIS 9.3
HSI-b	Hue-saturation-intensity (HSI) is a transformation of backscatter (proxy for seafloor hardness/softness), initially developed to decrease noise in radar reflectance [62]. Since backscatterrepresents seafloor reflectance, a HSI transformation may improve the separation of high andlow frequency signal scattering properties of the substratum.	ENVI 4.2
Maximum Curvature	Maximum Curvature provides the greatest curve of either the profile or plan convexity relativeto the analysis window [63].	ENVI 4.2
Rugosity	Rugosity provides the ratio of surface area to planar area within the analysis window and is to represent a measure of structural complexity [64].	Benthic Terrain Modeler Tool for ArcGIS

#### Generalized additive models

The GAM is an extension of GLMs, allowing several transformations to be applied to individual independent variables before addition to the model. This improves the ability of the model to deal with nonlinear data. The GAMs were implemented using the *R* ‘gam’ package within MGET. Where necessary local spline smoothers equivalent to two degrees of freedom were used [69]. Backward stepwise procedure was again used to determine variable importance based on the AIC.

#### Maximum entropy

The MAXENT approach has emerged as a powerful and flexible alternative to GLM and GAM for assessing species habitat suitability (see [14]). This general-purpose machine learning approach is designed for modeling species distributions based on presence-only data to determine the largest spread (i.e. maximum entropy) in a geographic dataset of species presences in relation to a set of background environmental variables [70]. This is essentially the same as maximizing the log likelihood of the data associated with the presence sites minus a penalty term [70]. This is conceptually similar to other commonly used information criteria (e.g. AIC). The penalty term regulates each environmental variable (known as a feature) by weighting it according to how much it adds complexity to the model; the sum of these weightings (including a regularization parameter, which is determined empirically) determines how much the likelihood should be penalized to prevent over-fitting [70]. Models used default settings; convergence threshold (0.00001), maximum iterations (1000), auto features, regularization multiplier (r = 1) and background points (10000).

### Data Treatment

The GLMs and GAMs were fitted using presence/pseudo-absence, while MAXENT used only the presence datasets from each survey method. For the towed-video-derived models a 1∶1 ratio of presence/pseudo-absence points were used (i.e. if there were 50 occurrences, then 50 pseudo-absence points were randomly generated; Table 3). For these towed-video datasets, pseudo-absence points were randomly generated along transects where no fish taxa were observed. For the baited-video-derived models pseudo-absences were generated from every deployment where the particular fish taxon was not observed (Table 3). All models used a set of relatively uncorrelated (i.e. spearman *rho*<0.5) multibeam sonar derived seafloor habitat variables as predictors (Table 4), and the fish occurrences as response variables (Table 3). For more detail on the 5 m^2^ cell resolution multibeam sonar and habitat variables see Monk et al. [31]. Habitat variables were log- or root-transformed as necessary to prevent extreme frequency distributions within GLM and GAM.

### Model Evaluation

Using the occurrence datasets that were set aside for model testing, model performance was evaluated using the threshold-independent AUC of the ROC (receiver operating characteristic) [71]. The ROC plots sensitivity (the fraction of occurrence records that are classified as presence) against 1 – specificity (the portion of absence points that are classified as absent) for all possible thresholds. A curve that maximizes sensitivity for low values of the false positive fraction is considered a good model and is quantified by calculating the AUC. An AUC value of 0.5 implies the model predicts species occurrence no better than random, and a value of 1.0 implies perfect prediction [71]. The ROC curves and the AUC values were calculated in DIVA-GIS.

### Potential Spatial and Temporal Confounding Factors

Semi-variograms and Moran’s *I* statistics were built using SAM (Spatial Analysis in Macroecology) to check all model residuals for spatial autocorrelation. Only very weak spatial auto-correlation (i.e. all taxa<0.1) was found and corrections were not needed [72].

It is accepted that the collection of the two datasets three years apart is not ideal. Research suggests that many of the nine species used were highly territorial and maintain strict territories year round (e.g. labrids, monacanthids and pempherids). Barrett [73], for example, studied the short- and long-term patterns of six temperate marine fishes (from labrid and monacanthid families) and found that these species appeared to be permanent residents of the reef. Accordingly, these nine fish taxa should exhibit similar niche characteristics over time. However, to statistically address the time discrepancies within the towed-video dataset a permutation analysis of variance (PERMANOVA; [74]) was used to test differences in the nine fish taxa between 2005/6 and 2009. The Bray-Curtis coefficient was used to create resemblance matrix. To account for undefined values caused by joint absences a dummy species value of 1 was added in all samples. The PERMANOVA was run with 9999 permutations to obtain P(perm) values under unrestricted permutation of raw data using time as a factor. No significant difference was detected between 2005/6 and 2009 for towed video (pseudo-*F*_1, 161_  =  2.28, P(perm) > 0.05). This evidence, combined with aforementioned research, indicates that time within and between video datasets is not a confounding factor.

### Similarity between Distribution Predictions

As proposed by Warren et al. [23], a modified Hellinger distance was used in order to compare between model predictions derived from the two observation techniques. This statistic (*I*) allows quantitative similarity assessments between distribution predictions (i.e. GIS grid layers) by computing the differences between them cell by cell. The *I*-values range from 0, indicating that the two predictions are completely different, to 1, suggesting that both are equal. The *I*-statistic is independent of sample size and predicted range sizes, making it superior to other metrics that have been proposed earlier [23]. This study adapted thresholds used by Roubicek et al. [24], and considered *I* values >0.8 are indicative of high degree of prediction overlap, values between 0.7 and 0.8 are moderate and values <0.7 indicate low similarity.
